# The Face-to-Face Still-Face (FFSF) Paradigm in Clinical Settings: Socio-Emotional Regulation Assessment and Parental Support With Infants With Neurodevelopmental Disabilities

**DOI:** 10.3389/fpsyg.2018.00789

**Published:** 2018-05-22

**Authors:** Lorenzo Giusti, Livio Provenzi, Rosario Montirosso

**Affiliations:** 0-3 Center for the at-Risk Infant, Scientific Institute IRCCS Eugenio Medea, Bosisio Parini, Italy

**Keywords:** early intervention, mother–infant interaction, neurodevelopmental disabilities, parents, still-face, rehabilitation

## Abstract

**Background:** The Face-to-Face Still-Face (FFSF) paradigm is a well-acknowledged procedure to assess socio-emotional regulation in healthy and at-risk infants. Although it was developed mainly for research purposes, the FFSF paradigm has potential clinical implications for the assessment of socio-emotional regulation of infants with neurodevelopmental disabilities (ND) and to supporting parenting.

**Aim:** The present paper describes the application of the FFSF paradigm as an evaluation and intervention tool in clinical practice with infants with ND and their parents.

**Methods:** Theoretical and methodological insights for the use of the FFSF paradigm in the clinical setting are provided. Single-case vignettes from clinical practice further illustrate and provide exemplifications for the use of the FFSF with infants with ND and their parents.

**Results:** From a clinical point of view, the use of the FFSF paradigm (1) offers a unique observational perspective on socio-emotional regulation in infants with ND and (2) enhances parents’ sensitivity to their infants’ behavior.

**Discussion:** The FFSF paradigm appears to be a useful tool for clinical assessment of socio-emotional regulation in infants with ND and promote the quality of parenting and early parent-infant interaction.

## Introduction

The Face-to-Face Still-Face (FFSF) paradigm (Tronick et al., 1978) is a well-known and validated procedure to assess socio-emotional regulation in infants facing a social stressor. The FFSF paradigm has been widely used with healthy and at-risk infants at different ages and it has contributed to improve our knowledge and conceptualization of early socio-emotional regulation development (Mesman et al., 2009). The suitability of this observational procedure to clinical settings has already been suggested (Miron et al., 2009). However, to the best of our knowledge, the FFSF has not been previously used to assess infants (and children) with a neurodevelopmental disability (ND; e.g., autism spectrum disorders, cerebral palsy, genetic syndromes) in a neurorehabilitation clinical context.

Our perspective is framed by the mutual regulation model (MRM; Gianino and Tronick, 1988). The MRM argues that the parent-infant interaction is organized by a bidirectional exchange of communicative signals that are used by the infant and the caregiver to coordinate the interaction and to cope with the stress of normally occurring interactive ruptures. From this perspective, the quality of the interaction is determined by the ability of each participant to cope with external stressors, regulate his/her emotional states, express communicative messages, and respond to his/her partner’s affective communications and regulatory needs. Caregivers’ behavior is guided by infants’ expressive displays (e.g., gaze, facial expressions, gestures, and vocalizations). In turn, infant behavioral and affective states are affected by the expressive displays of the caregiver. Importantly, the MRM – rather than emphasizing just synchrony – highlights that mother–infant interaction is a process characterized by matching and mismatching and that reparation of mismatches is a key developmental mechanism (Tronick and Beeghly, 2011).

Early caregiver–infant relationship may be strongly affected by the presence of a ND and many challenges arise for parenting in the contexts of infants’ disability. On the one hand, the parents face an augmented emotional burden (Dykens, 2015). On the other hand, infants with ND are less responsive and attentive, make fewer vocal and affective signals, are fussier, and produce less clear social cues (Okimoto et al., 2000). Moreover, infants (and children) with a ND have limited regulatory abilities and, therefore, they need their caregivers’ regulatory scaffolding to maintain emotional regulation and cope with interactive stress (Hauser-Cram and Woodman, 2016). Overall, these factors can disrupt the development of the functional dyadic co-regulatory system – including the reparation of mismatched process – and might further impact on infants’ socio-emotional regulation and parenting behavior. Additionally, it should be highlighted that infants with ND need to be hospitalized with their parents to take part in early rehabilitation programs (i.e., speech therapy, physical therapy, and so on). These programs recognize the importance of supporting parent-infant interaction as part of their daily work, but this focus is not widely implemented in practice (Innocenti et al., 2013). Moreover, having a broad picture of the infant functioning and parent–infant interactive patterns can be useful in the promotion of parenting skills.

From this perspective, we propose that the FFSF paradigm may be effectively used in clinical neurorehabilitation settings. Specifically, the FFSF provides a unique observational procedure for the assessment of infants’ socio-emotional regulation and the quality of early interaction in infants with ND and their parents. Importantly, according to a collaborative consultation approach (Boukydis, 2012), we used the FFSF videotapes to offer caregivers a brief parenting intervention aimed at enhancing parents’ sensitivity and responsiveness to their infant by helping them to observe and reflect upon the infant’s behavior.

Therefore, the general goal of the present paper is to provide a theoretical framework and methodological insights to use the FFSF in clinical settings specialized in the assessment of infants with ND and early parenting intervention. First, we provide a theoretical rationale integrating evidence from the FFSF paradigm research field and literature on socio-emotional regulation and parenting associated with ND. Second, we provide methodological insights for the application of the FFSF paradigm as a valid tool in the clinical setting dealing with ND, both for assessment purposes and parents’ support. Finally, we present clinical examples from our clinical practice highlighting the unique contributions of the FFSF procedure in this context.

### The FFSF Paradigm: A Privileged View on Infants’ Socio-Emotional Regulation Development and Parenting Behavior

The ‘70s have been a period of wide-spread growth for the infant research field (e.g., Stern and Stern, 2012; Sander et al., 2014). Within this field of research, the FFSF paradigm was developed by Tronick et al. (1978) to test the hypothesis that infants are active contributors in social interactions and to evaluate how they respond to the violation of interactive and social contingencies in the relationship with their main caregiver. In the FFSF paradigm, caregiver and infant engage in a 2-min-long face-to-face interaction (i.e., Play episode). Subsequently, the caregiver is instructed to stop any communication directed to the infant and to maintain a still face while keeping eye contact with the infant for 2 min (i.e., Still-Face episode). The lack of a contingent caregiver response is stressful for the infant who exhibits the typical *still-face* effect, which includes heightened negative emotionality as well as a reduction of social engagement and display of avoiding behaviors (Weinberg and Tronick, 1996; Weinberg et al., 1999; Montirosso et al., 2015a; Provenzi et al., 2015a). After the still-face exposure, the caregiver and the infant resume normal face-to-face interaction for another 2 min (i.e., Reunion episode). During the Reunion episode, a *carry-over* effect is usually observed, which consists in the persistent exhibition of negative emotionality and distress signals (e.g., avoiding behaviors) during the very first moments of the interaction (Weinberg and Tronick, 1996). Usually, during the Reunion, the mother–infant dyad gradually reaches a new homeostatic equilibrium and regains reciprocal positive interaction and playful exchanges (Montirosso et al., 2015a).

The FFSF paradigm has been used to assess developmental trajectories of infants’ emotional regulation (Hsu and Jeng, 2008; Yato et al., 2008), individual differences in behavioral stress response (Weinberg et al., 1999; Montirosso et al., 2015b), socio-cognitive domains such as episodic memory (Montirosso et al., 2013, 2014) as well as physiological reactivity (Muller et al., 2015; Provenzi et al., 2015b; Montirosso et al., 2016a,b; Provenzi et al., 2017). Moreover, the FFSF paradigm has also been used to investigate socio-emotional regulation in developmental risk conditions, such as preterm infants (Segal et al., 1995; Montirosso et al., 2010, 2016b), infants of depressed or anxious mothers (Weinberg et al., 2006; Kaitz et al., 2010; Reck et al., 2013).

Moreover, the interactive nature of the FFSF also allows researchers to assess parenting contribution to infants’ socio-emotional regulation. Different characteristics of parental interactive behavior have been found to be supportive of better socio-emotional regulation in infants: maternal sensitivity associates with more positive emotionality during the Still-Face episode (Braungart-Rieker et al., 2001; Chow et al., 2010); maternal social engagement associates with better regulation and less distress signals in the infant (Lowe et al., 2006; Montirosso et al., 2015b). Recently, one study has documented that infant and maternal behavior during normal face-to-face interactions (e.g., the Play episode) are both significant predictors of the infant’s ability to deal with socio-emotional stress (e.g., the Still-Face episode) at 6 months (Provenzi et al., 2016c).

### Socio-Emotional Regulation and Parenting Behavior in Infants With ND

Although they may present very different etiological, genetic and phenotypic characteristics, infants with ND often share a common pattern of difficulties in socio-emotional and behavioral regulation (Hauser-Cram and Woodman, 2016). Socio-emotional dysregulation has been reported in infants and children with cerebral palsy (Odding et al., 2006; Sigurdardottir et al., 2010), children diagnosed with an autism spectrum disorder (Mazefsky and White, 2014; Berkovits et al., 2017) as well as infants with genetic syndromes (e.g., Williams syndrome, Einfeld et al., 2001; Down syndrome, Jahromi et al., 2012). Previous FFSF research further highlighted difficulties in socio-emotional regulation in infants with ND. For example, the response of 3–13-month-old infants with Down syndrome lacked the typical reduction of positive emotionality to the Still-Face episode (Carvajal and Iglesias, 1997). Moreover, preschool children with a diagnosis of autism spectrum disorder reported the typical *still-face* effect but exhibited immature regulatory behaviors compared to age-matched typically developing children (Ostfeld-Etzion et al., 2015). Importantly, FFSF research also showed that parental behavior contributes to socio-emotional regulation even in infants with ND: in children with an autism spectrum disorder, maternal regulatory support was found to have a buffering effect on infants’ stress reactivity (Ostfeld-Etzion et al., 2015).

Furthermore, it should be noted that parenting assumes a different significance when an infant and child is diagnosed with a ND. Different dimensions of parenting are affected by the presence of a ND in infants, for example the ability to read the infant’s signals, to respond contingently and to provide adequate stimulations and sustain infants’ attention (Landry et al., 2008; Innocenti et al., 2013). Parents may face critical emotional burden (Dykens, 2015) and the acceptance of their own infant’s diagnosis may end up in emotional disturbances, such as high and chronic levels of distress, depression and anxiety (Baird et al., 2000; Papaeliou et al., 2012; Bemister et al., 2015; Cianfaglione et al., 2015; Cohrs and Leslie, 2017). Parents who have unresolved feelings about their infants’ ND also appear to be less able to provide regulatory support (Marvin and Pianta, 1996) and the reduction in maternal sensitivity has been found to be a key predictor of later socio-emotional development of their infants and children (Azad et al., 2013). At the same time, infants with ND may only partially give intelligible signals of their emotional states and needs (Okimoto et al., 2000). The difficulty in parental interpretation and appropriate responsiveness to infants’ cues and communications may further lead to the emergence of directive (Guralnick et al., 2008) or intrusive parenting style (Venuti et al., 2009; Bornstein et al., 2012; Blacher et al., 2013). Globally, these findings show that parent–infant relationship in the context of ND has a heightened risk of incurring in dysfunctional interactive transactions.

### Early Parental Interventions in Infants With ND

In light of this evidence, parenting behavior appears to play a key role in supporting infants’ socio-emotional development, even in the presence of ND. Early interventions that engage parents in assessment and rehabilitation phases have better long-lasting effects on developmental trajectories of infants and children with ND (Spittle et al., 2015). Additionally, investing in early interventions is also beneficial from a socio-economic point of view, as it guarantees a major economic return for healthcare systems (Doyle et al., 2009). Previous research that aimed to support parenting in families of infants with ND by using collaborative consultation on videotaped parent–infant interactions (Kim and Mahoney, 2005; Phaneuf and McIntyre, 2007; Lam-Cassettari et al., 2015) reported better outcomes for parental sensitivity and attunement as well as for infants’ behavioral stability and development. In recent years, an increasing number of intervention studies have used the video-feedback method in which infant behavior observation is performed with parents by a trained consultant (Juffer et al., 2012). Different approaches to the video-feedback are described in literature with the common goal of enhancing parental sensitivity and better behavioral strategies (for a review, Fukkink, 2008). The joint observation focuses on the observable interaction between parent and infant and has found to be effective in helping parents to recognize signs of infant’s stress, socio-emotional regulation strategies and parenting behavior also in dyads of infants with ND (Phaneuf and McIntyre, 2007; Poslawsky et al., 2015). The appropriateness of using FFSF paradigm within video-supported collaborative consultation with parents has been already suggested. For example, Papousek (2007) reported that using FFSF with parents can be effective in “*recruiting the parent’s intuitive competence and restoring intersubjective emotional relatedness as a basis for the infant’s recovery of communicative growth*” (p. 265). However, no previous study has specifically addressed the use of FFSF as an observational procedure for clinical assessment of infant socio-emotional regulation and the quality of early interaction in infants with ND and their parents.

## The FFSF Paradigm in Clinical Practice

### Subjects and Setting

We use the FFSF paradigm with 4–36-month-old infants diagnosed with a range of ND including cerebral palsy, autism spectrum disorders, genetic syndromes, and other mental retardation conditions. This age range is consistent with previous FFSF applications in research (Mesman et al., 2009; Provenzi et al., 2016) and the manipulation of maternal responsiveness is a reasonable age-appropriate stressor. Importantly, despite the fact that FFSF procedure has been mainly used with infants less than 10 months of age (Mesman et al., 2009), limited exceptions apply to the application of this paradigm to older infants with ND (Ostfeld-Etzion et al., 2015). Moreover, it should be noted that Montirosso and Tronick (2008) and Weinberg et al. (2008) have proposed a slightly modified version of the FFSF paradigm to be used with older infants up to 30 months of age.

The FFSF procedure usually takes place in a double-room with a unidirectional mirror and with a double-cam recording system: one camera is focused on the infant and the second one on the parent. In clinical settings, adaptations of the original FFSF paradigm should be done according to different infants’ variables: age, presence of comorbidity, motor impairment, severity of the disability. For example, with very young infants (<6 months of age) and with children who have severe motor disability and impairment, the infant may be placed in the infant seat or in the stroller as in the classical FFSF paradigm. However, with older children, it might be more appropriate to let the dyad interact on a carpet (Montirosso and Tronick, 2008; see also section “Case 2” below). Additionally, some infants with severe behavioral and/or cognitive impairments may require a longer warm-up period prior to the Still-Face episode, so that a longer Play episode could be set up. Obviously, due to the wide variety of clinical features among infants with ND, the choice of the different adaptations are generally based upon the clinician’s expertise and should be tailored on each infant’s characteristics.

#### Clinical Developmental Psychology Service

We are developmental and clinical psychologists working in a psychological service (i.e., *0-3 Center for the at-Risk Infant*) in a children rehabilitation institute located in northern Italy. Infants are hospitalized with their mothers for several days and they take part in a diagnostic and/or rehabilitation daily program (i.e., speech therapy, physical therapy, and so on). During the hospitalization, a multi-professional team follows infants and their parents with family-oriented approach. Usually, we combine structured (i.e., FFSF) and unstructured clinical observations (i.e., free play) in order to get, as much as possible, a global and informative picture of infants’ functioning and parent–infant interactive patterns. The cases presented in the current paper come from a pilot study which is part of a research project focused of the efficacy of early parenting intervention in families of infants with ND. The study protocol was approved by the Ethical Committee of the Scientific Institute IRCCS Eugenio Medea (Bosisio Parini, Lecco, Italy). All parents signed an informed consent.

### FFSF Paradigm to Assess Socio- Emotional Regulation in Infants With ND

The FFSF paradigm is micro-analytically coded for research purposes, however, this procedure is usually quantitative and time-consuming. In fact, in clinical settings it is usually preferred to have a less time-consuming qualitative evaluation. Nonetheless, although a specific training in formal coding system may not be necessary for clinical evaluations (Miron et al., 2009), we suggest that expertise on both FFSF procedure and atypical socio-emotional development of ND infants should be required, since age and developmental abilities should be taken in great consideration while interpreting infant behavior during the FFSF procedure. In the next paragraphs, some exemplificative – although non-exhaustive – elements are provided to guide clinical observation of the FFSF procedure. The Play episode, as a common face-to-face interaction, could inform about infants’ attention (see section “Case 2” below), social responsiveness, and emotional expressiveness (see section “Case 1” below). The Still-Face episode might provide specific information on infants’ sensitivity to maternal unresponsiveness (see section “Case 2” below) and on how he/she regulates emotional states (i.e., self- and other-directed behaviors; see sections “Case 1 and Case 2” below). Finally, the Reunion episode provides insights about infants’ ability to use parental support to recover from stressful experiences and to remember the previous interactive rupture (i.e., *carry-over* effect of negative emotionality; see section “Case 2” below). Moreover, specific features of parenting behavior may also be observed during normal interaction episodes (i.e., Play and Reunion). For example, insights can be obtained for what pertains to caregiver’s quality and intensity of emotional expressiveness (i.e., verbal and non-verbal), the quality of physical touch and emotional closeness to the infant (see section “Case 3” below), as well as sensitivity and responsiveness to infants’ signals and the emergence of directive (see section “Case 3” below) or intrusive interactive patterns. As for the Reunion episode, it might be enlightening to observe the caregiver’s ability of being supportive to infant’s regulation and the use of verbal expressions that convey the acknowledgment and recognition of the infants’ emotional and psychological inner world (i.e., reflective functioning). Finally, relevant information on specific dyadic coordination characteristics (i.e., matching/mismatching and reparation of mismatches) can be obtained during normal interaction episodes (i.e., Play and Reunion).

### FFSF Paradigm to Support Parenting in Infants With ND

Miron et al. (2009) pointed out how jointly reviewing tapes with caregivers provides an opportunity to inquire about how parents interpret their own and their infant’s behavior and about caregivers’ emotional responses in the interaction, both at the time of the procedure and in the *here and now*. Thus, involving parents in the viewing of the video-taped FFSF interaction might be an effective way to improve parental awareness of infants’ difficulties, to better learn to read and interpret infant’s signals and cues and to develop more appropriate parenting strategies (see sections “Case 3 and Case 4 below).

To set up a video-tape discussion session, a clear and short briefing is provided as follows: “*Now I would like to have a shared look of the videotape we made. You might be curious about some aspects of your infant’s behavior or you may have doubts about the best way to support his/her emotional and behavioral regulation. Let’s see if we can find useful information from this video. I will play the video and whenever you see something that you want to comment on, please feel free to intervene. I will do the same when I see something that catches my attention and curiosity*.” The focus on “parental curiosity” is crucial to avoid sending the message that the parent is going to be judged by the psychologist (Boukydis, 2012). The clinician should guide the discussion in a non-directive way, supporting the parents’ view and – when appropriate – suggesting new insights. During the joint observation of the FFSF videotape with parent, we often adopted the use of reflective comments (or aloud questions) such as “*I wonder why he/she here turned his head away?*” or “*What were his feelings at that moment, when you stopped interacting with him?*” Sometimes, infants and parents’ behaviors during the FFSF procedure are indicative of something that happens in their everyday life. In this case, parents should be encouraged to provide examples when they describe what they see in the videotape so that a connection is created between the current observation in the clinical setting and daily challenges at home.

## Clinical Examples

In order to give didactical exemplifications, here we provide some vignettes which have been elaborated based on actual clinical cases. These vignettes are meant to illustrate how the FFSF paradigm could be used: (a) to integrate the assessment of socio-emotional regulation in infants with ND disability (Cases 1 and 2), and (b) to support parental caregiving through a collaborative consultation using the video-feedback method with the *joint observation* of the FFSF interaction (sections “Case 3 and Case 4”).

### FFSF Paradigm to Assess Socio-Emotional Regulation in Infants With ND

#### Case 1

##### Clinical presentation

Peter and his family arrived to our clinical service when he was 14 months old. Perinatal anamnesis documented full-term birth after cesarean section with adequate extra-uterus adjustment, risk for insulin-dependent diabetes mellitus and reduced cranial diameters and good general health conditions at discharge from the Neonatal Intensive Care Unit (NICU). At 1 month, the analyses confirmed the significant reduction in brain volume. A diagnostic follow-up at 5 months documented the onset of epileptic seizures. Overall, Peter appeared to be easily dysregulated by moderate-to-high intensity stimulations and poor emotional regulation were reported.

##### Aim

To obtain more specific and fine-grained information on Peter’s emotional regulation during a standardized observational procedure.

##### The FFSF paradigm session

During the Play episode, Peter was specifically sensitive to tactile (e.g., mother caressing) and auditory (e.g., mother singing) stimulations. In response to this kind of stimulations, Peter showed positive emotionality (e.g., rhythmic movements of the chest and arms together with wide mouth opening). During the Still-Face episode, Peter immediately appeared alert and sensitive to the suspension of maternal communication by exhibiting an immediate stress response (i.e., increase of motor activity and emergence of disorganized movements of the chest and arms). Interestingly, specific patterns of dyadic matching behaviors emerged including attempts to reach out with the arms directed to the mother’s body while simultaneously seeking eye-contact with her in order to be picked up. Such relational regulatory strategies were something that Peter usually did not show when distressed, as he easily became disorganized.

##### What does the FFSF add to clinical assessment?

During the maternal unavailability episode Peter shows a clear ability to regulate his own behavior and emotions through relational strategies, something otherwise non-observable with a different observational procedure. Although such regulatory strategies may not be usual for the infant, it is clinically relevant to highlight that in response to specific relational and environmental adjustments (i.e., face-to-face interaction, lowered stimulation, enough time to organize other-directed attempts to self-regulate) Peter can access to more adaptive strategies to achieve regulation. This knowledge enriches the picture of Peter difficulties in self-stabilization and highlights a dyadic pattern to reinforce in order to support infant’s socio-emotional regulation.

#### Case 2

##### Clinical presentation

Eleanor arrived at our clinical service with her family at 26 months of age for a behavioral evaluation. She had received a diagnosis of autism spectrum disorder with severe mental retardation (Development Quotient = 50; Bayley scales, Bayley, 1993) 2 months before. The neuropsychiatrist who referred the case was worried about the apparent total absence of social and relational engagement of the child, even when the parent or the psychologist tried to elicit any response.

##### Aim

To explore Eleanor’s sensitivity to the manipulation of maternal responsiveness.

##### The FFSF paradigm session

Due to Eleanor’s age, the carpet setting was chosen. The father decided to participate in the FFSF paradigm with his daughter. Moreover, we decided to use a modified FFSF paradigm (i.e., prolonged 4-min-lasting Play episode) to give the infant more opportunities to exhibit some relational behavior. During the Play episode, Eleanor’s attentional focus was mainly on objects (e.g., a toy-car) with very limited gazes to the father. The father pushed the toy car several times and when it hit the wall Eleanor produced screams of excitement without looking at the parent. When the Still-Face episode started, the father had already pushed the car for the umpteenth time. The car hit the wall and Eleanor showed the usual reaction. Nonetheless, as the father was not communicating with the infant in any way, after 30–40 s Eleanor produced intentional vocalizations directed to the father, approached him physically, exhibited a brief gaze directed to his face and attempted to hug the parent. Interestingly, when the Reunion phase started, the father tried to re-engage the child in the usual toy car game, but she seemed to be less interested and produced some negative emotional vocalizations (i.e., carry-over effect).

##### What does the FFSF add to clinical assessment?

The use of the FFSF paradigm with Eleanor and her father allowed to observe something unexpected. First, it is certainly true that the lack of social responsiveness is a core feature of autism spectrum disorder and of Eleanor’s functional behavioral organization. Still, it was evident that when the rupture in father–infant interaction occurred, Eleanor started displaying more intentional and other-directed communicative behaviors to re-engage the father and to obtain physical proximity. Moreover, during the Reunion episode Eleanor also showed to the primary activation of a motivational system related to bonding and comfort that overcame the need and the pleasure to engage in ritualized play scripts. While taking into account her relational and communicative deficit, Eleanor showed an unexpected pattern of other-directed behavior in response to the caregiver’s interactive change. In this case, the FFSF allowed to focus Eleanor’s residual interactive competencies showing her contribution in mutual regulation processes. Here, the FFSF highlighted fine-grained information on dyadic matching and mismatching states, as well as the attempts of reparation performed by the child.

### FFSF Paradigm to Support Parenting in Infants With ND

#### Case 3

##### Clinical presentation

Nicholas is a 5-month-old infant who was transferred to our Institute after 4 months of NICU hospitalization in the community hospital. He suffered from a severe neonatal injury with several cerebral lesions due to a prolonged hypoxia at birth. Nicholas’ parents reported sad feelings and anger for the infant’s condition. Moreover, they also found no pleasure in the interaction with Nicholas and they reported that, despite their attempts to stimulate him, he was totally unexpressive and unresponsive in daily interactions.

##### Aim

To discuss with the mother on these topics: (1) increasing maternal acknowledgment of Nicholas’ regulation needs and communicative signals; (2) promoting more adaptive ways of interacting with him.

##### The FFSF paradigm

During the Play episode, the mother talked loudly, moved the baby’s hands with a directive style and repeatedly invited him to engage in singing together. Nicholas alternated fussiness and self-absorbed behaviors (i.e., lack of social responsiveness). During the Still-Face episode, Nicholas responded with the following behavioral sequence: (1) self-absorbed behaviors along with few rapid gazes directed to the mother, (2) rapid increase in fussiness and cry and behavioral disorganization, (3) a gradual stabilization with a prolonged state of mother-directed attention and positive emotionality while looking at the mother. As soon as the Reunion episode started, the mother rapidly tried to re-engage with Nicholas with the same high-energy and physical stimulations that characterized the Play episode. Nicholas reacted to these stimulations by becoming fussy and avoidant.

##### The FFSF implications for parent support

The consultation with Nicholas’ mother had some pivotal moments. First, when the mother looked at Nicholas clearly smiling at her during the Still-Face episode she seemed to be surprised and she said “*I can’t believe it. I didn’t catch that during the session.*” This emergent state of curiosity and surprise about her infant’s communicative behaviors allowed the psychologist to work with the parent on her mental representation of Nicholas’ socio-emotional regulation functioning. Secondly, when the mother was watching the Reunion episode, the psychologist said: “*I wonder why he is getting fussy again.*” This open question facilitated some exchanges with the mother on the better ways to interact with Nicholas. Some key points emerged, such as the importance of (1) reducing the quantity and energy of physical contacts, (2) increasing the use of visual cues and emotional facial displays, (3) leaving more silent moments to allow Nicholas to achieve a stable behavioral state and to be more available to interact.

##### What does the FFSF add to parenting support?

During the consultation, Nicholas’ mother was able to explore specific aspects of the interaction with the infant and better ways to repair interactive ruptures. In particular, she achieved a deeper acknowledgment of the relevance of his communicative behaviors and regulatory needs and she got access to more adequate ways to engage with him.

#### Case 4

##### Clinical presentation

Sarah was 8 months old when she first came to our clinical unit with her family. Sarah had a severe visual impairment. Moreover, Sarah’s mother reported that she faced many challenges in trying to understand her daughter and interacting with her in a reciprocal satisfying way. The visual impairment of the infant was a great barrier for the mother, who reported feelings of sadness and inadequacy as a caregiver.

##### Aim

To support the mother in detecting communicative intent and participation in Sarah’s behavior during the interaction and to increase the opportunity for the dyad to perceive more pleasure in their relationship.

##### The FFSF paradigm

We videotaped the FFSF paradigm with Sarah and her mother. During the Play episode, Sarah demonstrated to be able to orient her attention to auditory stimuli (e.g., maternal voice), but the mother rarely interpreted and responded properly to her orientation as communicative signals. The mother was frequently silent producing few attempts to engage with Sarah and remaining physically distant. The mother used the pacifier any time Sarah displayed minimal distress. The interaction appeared to be very difficult and – according to maternal reports – not very satisfying. During the Still-Face episode Sarah stood relatively quiet, with very few movements of the arms and legs. Nonetheless, a few seconds after the Still-Face episode onset she started to protrude in the direction of the mother, producing a lot more vocalizations compared to the Play episode and showing increased distress and negative emotionality. As soon as the Reunion episode started, the mother resumed to interact with Sarah and said: “*Oh, you’re here then!*” During the reunion phase the mother appeared more active in the interaction with her daughter and, after a period of negativity (i.e., 3040 s), Sarah displayed a smile toward her mother.

##### The FFSF implications for parent support

During the consultation the Sarah’s mother immediately said that she had never seen Sarah being so communicative and that the observation procedure “*literally opened a new window in our relationship.*” Looking at Sarah’s response to the Still-Face episode further allowed the mother and the psychologist to identify specific communicative behaviors which were previously undetected by the mother (e.g., active requests of being picked up, attentional focus on the mother, and sensitivity to maternal lack of communications). Moreover, the mother acknowledged that Sarah was indeed interested in interacting with her even in the presence of visual impairment. This increased acknowledgment of Sarah’s availability and desire to interact, and was also an opportunity to set up a collaborative consultation on specific modalities to reach out to Sara and to achieve better emotional closeness and reciprocal satisfaction.

##### What does the FFSF add to parenting support?

This clinical vignette suggests that the joint observation of FFSF promote in the Sarah’s mother some changes in mental representation of her own infant. In turn, it reflects on subtle modifications in the maternal interactive style during interaction with Sarah.

## Limitations

Some limitations in the present work should be acknowledged. First, the present paper should be considered a clinical report and as consequence it has limited generalizability. This kind of study design appears to be adequate to corroborate evidence from clinical experience. In the future, clinical trials are needed to assess the effectiveness of the FFSF paradigm in clinical settings with infants diagnosed with ND and their parents. Second, infants with ND disabilities may present wide individual differences in their socio-emotional response to the FFSF paradigm and the same applies to their caregivers’ behavior. Clearly, infant’s characteristics and parenting could be different according to clinical conditions and to the degree of disability and psycho-motor impairments. For example, the use of the FFSF procedure might not be feasible with infants who present a very severe ND condition. Third, it is worth noting that the use of FFSF in clinical setting with atypically developing infants implies to take in consideration potential ethical issues (Schneider et al., 2000). Although a great number of FFSF studies have documented that infant distress associated with maternal unavailability is well tolerated by infants, professionals and researchers must remain mindful of ethical issues that may be involved in using the FFSF paradigm. Thus, while early intervention practitioners should use non-stressful methods to understand infant’s socio-emotional skills, the FFSF should be used for clinical purposes only for the assessment of specific infant’s characteristics or aspects of the parent–infant interaction (i.e., socio-emotional regulation and/or dyadic coordination). This precaution is critical to avoid exposing the infant to unnecessarily overwhelming stress. Finally, we recommend that the use of this observational procedure in clinical settings should be pursued only by practitioners who have expertise in the use of the FFSF procedure and in the behavioral assessment and parental support with ND infants.

## Conclusion

The FFSF is a tool that can help practitioners assess the infant’s socio-emotional regulation competence and to evaluate parenting behaviors. The use of the FFSF in clinical neurorehabilitation settings for young infants with ND and their parents holds the potential of providing additional aspects of infant/child’s functioning and parent–infant interactive patterns. Also, the use of FFSF using the video-feedback method allows early intervention practitioners to provide parents with a common ground for collaborative consultation based on the infant’s behavior and the interactive patterns of the parent–infant dyad. To this regard, although in the present paper we discussed separately infants’ socio-emotional regulation skills and parenting behaviors in clinical vignettes, we acknowledge that the majority of highlighted behaviors and processes are inherently dyadic in their nature. The FFSF is framed by a theoretical perspective of caregiver–infant early interaction as organized by a bidirectional exchange of communicative signals that are used by the infant and the caregiver to coordinate the interaction and to cope with the stress of inevitable interactive ruptures (i.e., MRM, Gianino and Tronick, 1988). This latter is even more important to support parents with infants with a ND in which the dyadic co-regulatory system is strongly affected by the presence of disabilities. A unique aspect of the FFSF is that the Reunion period is a special time for the caregiver and the infant to repair the interaction following a prolonged interactive disruption. Thus, the use of the FFSF paradigm for clinical purposes in the case of our young patients and their mothers allows to observe individual differences in socio-emotional regulation and dyadic reparation during a well-validated, age-appropriate social stress procedure. Overall, our clinical cases add to the rationale of using this observational procedure to identify specific dyadic behaviors that may be important in clinical settings with atypically developing infants and their parents.

At a very general level, the use of a laboratory procedure such as FFSF in the clinical setting is in line with the translational approach in the scientific research. That is, the efforts of harnessing knowledge from basic sciences to implement new treatment options for patients (Woolf, 2008). In infants with ND this effort is essential to improve the quality of life of infants and their parents, given that this provides knowledge and techniques generated in infant research which can promote new approaches for infant assessment and parental intervention. We suggest that the use of the FFSF procedure might help address this issue, highlighting that a more direct focus on parenting should be considered as part of early effective interventions in neurorehabilitation programs. Consistently, we hope that the current paper will provide a contribution for practitioners that will promote the intervention on parenting as a key aspect of family-centered practice.

## Author Contributions

All co-authors contributed to the present manuscript. LP conceived the main idea for this work. LP and LG wrote the initial draft of the manuscript. RM provided supervision and helped with the final version of the manuscript. All the authors approved the submission of the present manuscript to Frontiers in Developmental Psychology.

## Conflict of Interest Statement

The authors declare that the research was conducted in the absence of any commercial or financial relationships that could be construed as a potential conflict of interest.
